# Transmission characteristics in Tuberculosis by WGS: nationwide cross-sectional surveillance in China

**DOI:** 10.1080/22221751.2024.2348505

**Published:** 2024-04-30

**Authors:** Dongxin Liu, Fei Huang, Yaru Li, Lingfeng Mao, Wencong He, Sihao Wu, Hui Xia, Ping He, Huiwen Zheng, Yang Zhou, Bing Zhao, Xichao Ou, Yuanyuan Song, Zexuan Song, Li Mei, Li Liu, Guoliang Zhang, Qiang Wei, Yanlin Zhao

**Affiliations:** a National Pathogen Resource Center, Chinese Center for Disease Control and Prevention, Beijing, People’s Republic of China; b National Tuberculosis Reference Laboratory, National Center for Tuberculosis Control and Prevention, Chinese Center for Disease Control and Prevention, Beijing, People’s Republic of China; c Department of Nutrition, Beijing Friendship Hospital, Capital Medical University; d Joint Research Center for Molecular Diagnosis of Severe Infection in Children, Binjiang Institute of Zhejiang University, Hangzhou, People’s Republic of China; e Laboratory of Respiratory Diseases, Beijing Key Laboratory of Pediatric Respiratory Infection Diseases, Beijing Pediatric Research Institute, Beijing Children’s Hospital, Capital Medical University, Key Laboratory of Major Diseases in Children, Ministry of Education, National Clinical Research Center for Respiratory Diseases, National Center for Children’s Health, Beijing, People’s Republic of China; f National Clinical Research Center for Infectious Diseases, Guangdong Clinical Research Center for Tuberculosis, Shenzhen Third People’s Hospital, Shenzhen, People’s Republic of China

**Keywords:** Tuberculosis, transmission, risk factors, MDR/RR-TB, whole genome sequencing

## Abstract

China, with the third largest share of global tuberculosis cases, faces a substantial challenge in its healthcare system as a result of the high burden of multidrug-resistant and rifampicin-resistant tuberculosis (MDR/RR-TB). This study employs a genomic epidemiological approach to assess recent tuberculosis transmissions between individuals, identifying potential risk factors and discerning the role of transmitted resistant isolates in the emergence of drug-resistant tuberculosis in China. We conducted a population-based retrospective study on 5052 *Mycobacterium tuberculosis* (MTB) isolates from 70 surveillance sites using whole genome sequencing (WGS). Minimum spanning tree analysis identified resistance mutations, while epidemiological data analysis pinpointed transmission risk factors. Of the 5052 isolates, 23% (1160) formed 452 genomic clusters, with 85.6% (387) of the transmissions occurring within the same counties. Individuals with younger age, larger family size, new cases, smear positive, and MDR/RR were at higher odds for recent transmission, while higher education (university and above) and occupation as a non-physical workers emerged as protective factors. At least 61.4% (251/409) of MDR/RR-TB were likely a result of recent transmission of MDR/RR isolates, with previous treatment (crude OR =   2.77), smear-positive (cOR =   2.07) and larger family population (cOR = 1.13) established as risk factors. Our findings highlight that local transmission remains the predominant form of TB transmission in China. Correspondingly, drug-resistant tuberculosis is primarily driven by the transmission of resistant tuberculosis isolates. Targeted interventions for high-risk populations to interrupt transmission within the country will likely provide an opportunity to reduce the prevalence of both tuberculosis and drug-resistant tuberculosis.

## Introduction

According to the 2022 WHO tuberculosis report, MTB is estimated to have caused 10.6 million new cases of tuberculosis (TB) worldwide in 2021 [1]. Amid the crisis, the rise of drug-resistant tuberculosis, especially rifampicin-resistant (RR-TB) and multidrug-resistant (MDR-TB) strains, poses a severe public threat to effective TB control worldwide. Halting TB and MDR/RR-TB transmission is crucial for the tuberculosis epidemic. Multiple factors can contribute to the tuberculosis epidemics [2–4], epidemiological research on tuberculosis transmission can provide insights into the local factors that drive tuberculosis transmission and multidrug-resistant or rifampicin-resistant tuberculosis (MDR/RR-TB) and provide scientific data to formulate effective intervention strategies [5].

China holds the third largest share of global tuberculosis cases; reports estimate 780,000 new cases in 2021, and the estimated number of MDR/RR cases was 33,000[6]. Previous studies have described the transmission patterns of tuberculosis or multidrug-resistant tuberculosis isolates from China using effective genotyping methods combined with enhanced epidemiological investigation [7–9]. However, these studies have often been confined to one city or province, limiting their relevance for understanding TB transmission at the national level. The factors influencing the transmission and emergence of TB and MDR/RR-TB remain understudied in China.

Measuring tuberculosis transmission and estimating recent transmission is exceedingly tricky, considering that infection in only a minority of individuals progresses to disease, and the latency periods among those who do progress are variable[10,11]. Recent advances in whole-genome sequencing (WGS) have significantly improved the determination of genetic relatedness between isolates and the identification of drug resistance. This study systematically collected and sequenced 5052 MTB isolates from 70 counties across all 31 provinces, along with related epidemiological information. We aimed to use this nationally representative dataset to assess genomically clustered tuberculosis transmission in China and identify related protective/risk factors. Meanwhile, we assessed the distribution of drug-resistant mutations in different clusters and, combined with treatment history, quantified the magnitude of drug-resistant TB arising from transmission. Based on this study, we hope that effective interventions can be formulated to interrupt TB transmission and facilitate TB control, mainly aiming to prevent and reduce the emergence of MDR/RR tuberculosis.

## Methods

### Study setting

China has consistently been amongst the countries burdened by a high prevalence of TB and drug-resistant TB. The Chinese CDC’s National Tuberculosis Reference Laboratory (NTRL) department conducted the survey using cluster-randomized sampling to obtain a representative overview of tuberculosis patients in China. Seventy drug-resistance surveillance clusters nationwide acted as sample sources (Supplementary Figure 1). The number of clusters assigned to each province was proportional to the number of new smear-positive cases reported by that province relative to the total number of cases nationwide in 2004 and 2005, ensuring that each province had at least one cluster. The primary sampling unit for cluster sampling was the local tuberculosis clinic at the county or district level, typically located at the local site of the CDC, providing outpatient tuberculosis service across China. Eligible patients were those presenting as presumptive tuberculosis cases newly registered during the survey period (from January to December 2013) at selected tuberculosis clinics, and all eligible patients were consecutively enrolled. A presumptive case was defined as a patient with a persistent cough for more than two weeks or at least two of the following symptoms: fever, drenching night sweats, unexplained weight loss (>1.5 kg/month), general feeling of illness (malaise) and tiredness, and shortness of breath with chest pain [12].

Two trained interviewers independently interviewed each enrolled patient, using a standard questionnaire to collect demographic data (gender, age, family population, nationality, occupation, and education level) and clinical data (new case or previously treated, diabetes, hepatitis, and smear microscopy). A third interviewer resolved the discrepancies in the interview data. The China CDC approved the study of the Tuberculosis Research Ethics Review Committee, and written informed consent was obtained from each participant. All authors vouched for the completeness and accuracy of the data presented.

### Specimen processing and sequencing

Two sputum samples for culturing were obtained from each eligible patient who can provide qualified sputum before the initiation of treatment. Each specimen was treated with one volume of 4% sodium hydroxide per one volume of sputum, followed by homogenization through vigorous stirring to isolate the culture. Subsequently, an aliquot of 0.1 ml from the resulting specimen was inoculated into two tubes of acidified Löwenstein – Jensen medium and the inoculated tubes were then incubated at 37°C. The cultures were assessed during the first week for rapidly growing bacteria and subsequent for slower growing bacteria every week, the result was negative if no bacteria were detected by the eighth week. Mycobacterial nucleic acid was extracted using the cetyl-trimethyl-ammonium-bromide method [13]. Genomic DNA was sequenced using the Illumina Hiseq-2000. Paired-end reads were aligned to the reference genome H37Rv (NC_000962·3) using the Burrows–Wheeler algorithm and sorted with SAMtools (V.1.15). Variant calling was conducted with GATK to identify single nucleotide polymorphisms, low-quality SNPs (Phred score Q < 20 and read depth < 5) and sites with missing calls in >10% of isolates removed from analysis. SNPs located within 12 bp of each other or that had less than 75% of supporting high-quality reads were also excluded. Variations in known drug-resistant genes or intergenic regions, direct repeat regions, microsatellite-like sequences, transposition insertion sequences (such as *IS6110*), ESX secretion system protein genes, and PE/PPE/PGRS family genes were masked for phylogeny analysis and minimum spanning tree reconstruction [8]. *Silico* drug resistance prediction was carried out as described previously [14].

### Transmission cluster identification based on genetic distance

Transmission clusters were defined by applying a threshold of 12 or fewer pairwise SNPs between sequences as previously specified [7]. The minimum spanning tree was generated with Phyloviz. In the primary analysis, isolates from the same county were considered local transmission. Otherwise, we defined it as cross-county transmission.

### Transmission and positive selection of MDR/RR isolates

Genomic clusters may represent the transmission of an MDR/RR strain or the initial transmission of a non-MDR/RR strain that later developed resistance. If isolates share the same resistant mutations within a cluster, we inferred that the emergence of drug-resistant tuberculosis was attributable to the transmission of drug-resistant isolates. Conversely, if the isolates within a cluster exhibited different genotypic resistance and were isolated from previously treated patients, we considered it indicative of resistance during transmission. The presence of MDR/RR-TB among treatment-naïve patients suggested the transmission of MDR/RR isolates.

### Statistical analysis

Categorical variables were expressed in numbers (percentages) and compared using the chi-square test. Univariate and multivariable logistic regression models were used to calculate the odds ratios (ORs) and 95% confidence intervals (CIs) for the risk factors associated with genomic clusters. A forward, step-wise approach was used to add covariates to the logistic regression model. Statistical significance was set at a 2-sided *P* value < 0.05. Data cleaning and statistical analysis were performed using SAS.

## Results

### Dataset description

We successfully sequenced 5,052 distinct *M. tuberculosis* isolates, meeting genomic quality standards (excluded polyclonal infections, low alignment to reference, and missing more than 10% of qualified SNPs). Four significant lineages of MTB were observed, with the majority coming from L2 (3,774 isolates) and L4 (1,242 isolates), while L1 (29 isolates) and L3 (7 isolates) comprised the remainder.

The prevalence of drug resistance in the dataset, as defined by molecular genotyping, was 6.77% for MDR TB. Among all enrolled patients with available epidemiological information, 72.92% were male, 66.7% were farmers, 92.83% belonged to the Han nationality, 44.01% had a primary education level or below, 25.68% lived in urban areas, and the mean age ± standard deviation (SD) was 46.97 ± 18.56 years. Additionally, 23.21% were previously treated for tuberculosis, 8.12% had previously been diagnosed with diabetes, 3.43% had hepatitis B, and 75.1% were smear-positive (Supplementary Table 1).

### Risk/protective factors of clustering of all isolates

**Figure 1.**
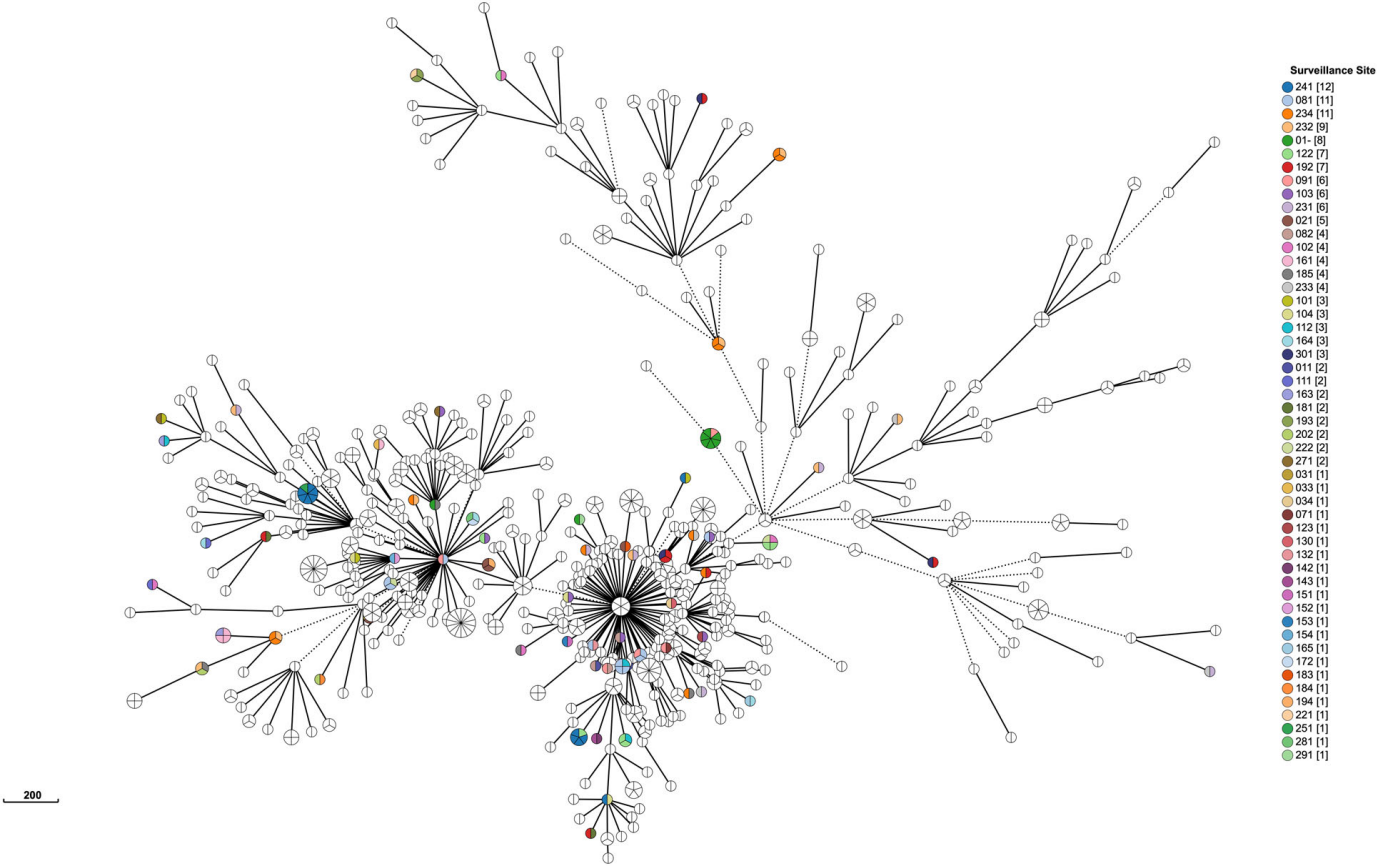
**Minimum spanning tree of all *Mycobacterium tuberculosis* isolates**. We defined genetic distances within 12 SNPs isolates were clusters, only clustered isolates were retained. Each circle represents a cluster. The size of the circle represents the number of isolates in the cluster. The colour of each circle represents the surveillance site of the isolates. Isolates isolated from the same county within a cluster were coloured by white but not mean all white colour isolates isolated from same site.

We identified 452 genomic clusters, ranging in size from two to fourteen isolates, involving 1,160 of 5,052 isolates, yielding a clustered proportion of 23.0% within the county. Among these clusters, isolates within 387 (85.6%) clusters were exclusively isolated from the same county; there were 65 cross-county clusters, comprising two to seven isolates, with a geographic distribution range of two to three counties (Figure 1 and Supplementary Figure 1 and Supplementary Table 2)

Table 1 displays the risk and protective factors associated with the likelihood of clustering. Risk factors included younger age (*P*-trend = 0.01), larger family size (adjusted OR  = 1.11 (95% CI, 1.07–1.15), *P *< 0.001), new tuberculosis cases (aOR = 1.48 (95% CI, 1.22–1.79), *P *< 0.001), smear-positive (aOR = 1.52 (95% CI, 1.26–1.83), *P *< 0.001) and MDR/RR (aOR = 1.59 (95% CI, 1.23–2.05), *P *< 0.001), while higher education (university and above) (OR = 0.57 (95% CI, 0.37–0.88), *P *= 0.011) and occupation as non-physical workers (aOR = 0.72 (95% CI, 0.54–0.95), *P *= 0.022) were identified as protective factors, using the ordered logistic regression analysis,.

**Table 1.** The risk/protective factors associated with the likelihood of clustering.

Characteristics	Cluster rate	Crude OR	*P*-value	Adjusted OR*	*P*-value
**Demographic factors**					
Gender					
Male	782 (22.29)	ref		ref	
Female	312 (23.94)	1.10 (0.95–1.28)	0.224	1.00 (0.84–1.18)	0.958
Age					
<20	86 (29.76)	ref		ref	
20–39	334 (23.08)	0.71 (0.54–0.94)	0.016	0.74 (0.51–1.07)	0.106
40–59	401 (23.49)	0.73 (0.55–0.96)	0.022	0.80 (0.55–1.17)	0.254
60–79	249 (20.46)	0.61 (0.46–0.81)	0.001	0.65 (0.43–0.97)	0.037
80+	24 (16.00)	0.45 (0.27–0.75)	0.002	0.52 (0.28–0.96)	0.037
*P*-trend		<0.001		0.01	
Current residence					
County	231 (19.85)	ref		ref	
Town	767 (22.77)	1.19 (1.01–1.41)	0.038	1.06 (0.85–1.33)	0.597
Family population size		1.12 (1.08–1.16)	<0.001	1.11 (1.07–1.15)	<0.001
Nationality					
Han	1022 (22.87)	ref		ref	
Other	72 (20.87)	0.89 (0.68–1.16)	0.392	0.79 (0.57–1.09)	0.156
Occupations					
Farmer	753(23.48)	ref		ref	
Student	56 (25.23)	1.10 (0.80–1.51)	0.554	1.05 (0.66–1.65)	0.85
^#^Non-physical workers	122 (16.8)	0.66 (0.53–0.81)	<0.001	0.72 (0.54–0.95)	0.022
Other (housekeeper or unemployed)	163 (24.96)	1.08 (0.89–1.32)	0.417	1.05 (0.82–1.36)	0.691
Education					
Primary and below	435 (21.79)	ref		ref	
Middle school	515 (22.99)	1.07 (0.93–1.24)	0.351	0.99 (0.82–1.19)	0.909
University and above	46 (15.38)	0.65 (0.47–0.91)	0.012	0.57 (0.37–0.88)	0.011
**Clinical factors**					
Diabetes					
Yes	68 (17.53)	ref		ref	
No	1017 (23.16)	1.42 (1.08,1.86)	0.012	1.31 (0.97–1.76)	0.076
Hepatitis B					
Yes	27 (18.00)	ref		ref	
No	936 (22.19)	1.30 (0.85,1.98)	0.225	1.37 (0.87–2.14)	0.17
Previous TB					
Yes	206 (18.44)	ref		ref	
No	888 (24.03)	1.40 (1.18–1.66)	<0.001	1.48 (1.22–1.79)	<0.001
Smear microscopy					
Negative	187 (16.45)	ref		ref	
Positive	877 (25.58)	1.75 (1.47–2.08)	<0.001	1.52 (1.26–1.83)	<0.001
**Bacteriological factors**					
MDR					
Yes	99 (28.95)	1.40 (1.10–1.79)	0.007	1.60 (1.21–2.10)	0.001
No	1061(22.53)	ref		ref	
Lineage					
1	4 (13.79)	ref		ref	
2	898 (23.79)	1.95 (0.68–5.62)	0.216	2.65 (0.91–7.75)	0.075
3	2 (28.57)	2.50 (0.36–17.57)	0.357	0.01 (0.01–99.9)	0.969
4	256 (20.61)	1.62 (0.56–4.70)	0.373	2.16 (0.73–6.35)	0.163

Abbreviations: OR, odds ratio; TB, tuberculosis; MDR, multidrug-resistant.

* The logistic model was adjusted for age, gender, current residence, family size, nationality, occupations, education, diabetes history, hepatitis B, previous TB, smear microscopy, MDR/RR, and lineage, except for the stratified factors.

^#^ Non-physical workers: commercial service personnel, enterprise staff, medical personnel, educational staff, civil servant and public institutional person.

### Transmission and acquisition of MDR/RR-TB

**Figure 2.**
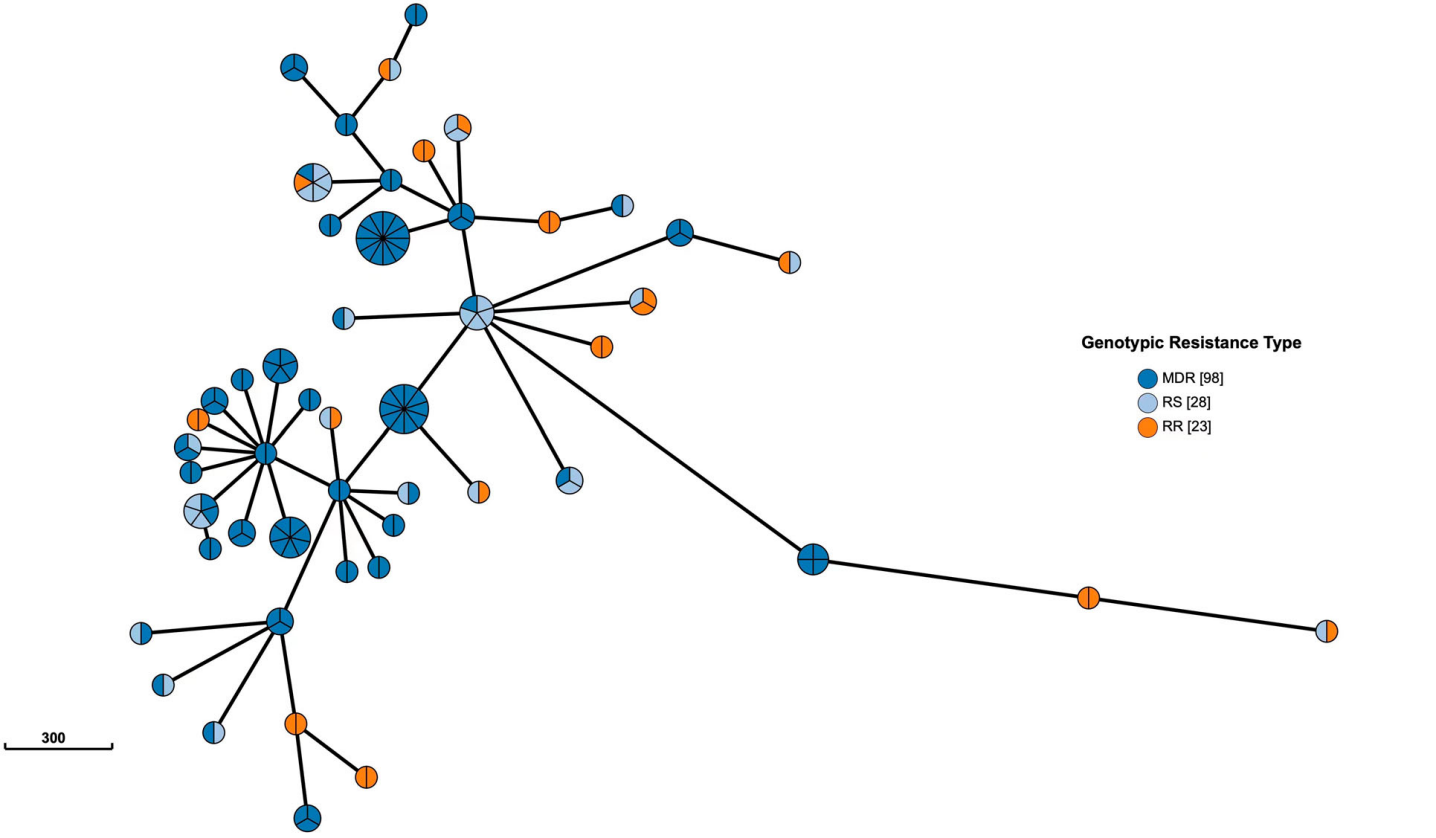
**WGS-clusters of multidrug resistant or rifampin-resistant isolates with rifampin-sensitive isolates**. 98 multidrug resistant (MDR) isolates, 23 rifampin-resistant (RR) isolates and 28 rifampin-sensitive (RS) isolates clustered in 50 WGS – clusters.

**Figure 3.**
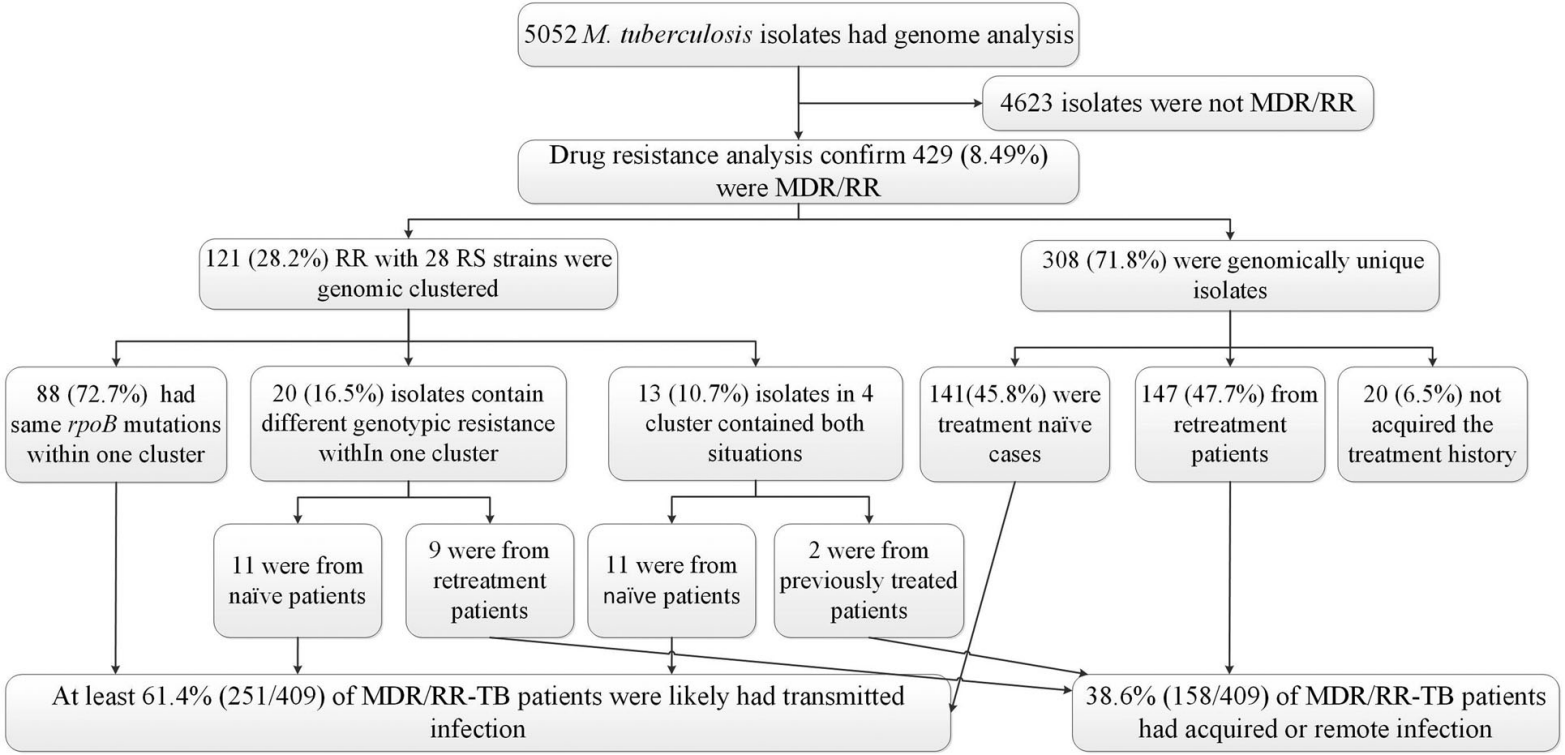
**Classification of MDR/RR-TB based on treatment history and genomic analysis**. MDR/RR-TB: multidrug resistant or rifampin-resistant tuberculosis.

We identified 429 MDR/RR isolates, of which 121 and 28 rifampin-sensitive isolates were clustered into 50 WGS clusters (Figure 2). Within these clusters, 32 contained 88 isolates with the same *rpoB* mutations within one cluster. Eight clusters, including 9 isolates, exhibited different genotypic resistance and were isolated from previously treated patients, suggesting the possibility of recently developed RR-related mutations from RS isolates. Another 9 clusters involving 11 RR isolates and also showed different genotypic resistance but were isolated from naïve patients, indicating transmission of MDR/RR isolates. 4 clusters (10, 29, 98, 111) contained both same *rpoB* mutations and different genotypic resistance, 11 isolates were caused by transmission, and 2 isolates may be from positive selection. 308 were genomic unique isolates, with 141 isolated from the treatment naïve cases, 147 from retreatment patients, and 20 without recorded treatment history excluded from analysis. Thus, at least 61.4% (251/409) of MDR/RR-TB patients were likely caused by the recent transmission of MDR/RR isolates in China (Figure 3).

### Risk/protective factors for MDR/RR-TB clustering

Table 2 shows the results of the univariate analysis of the risk/protective factors associated with genomic clustering of MDR/RR-TB. Previous treatment (crude OR =   2.77 (95% CI, 1.74–4.42), *P *< 0.0001), smear-positive (cOR =   2.07 (95% CI, 1.06–4.04), *P *= 0.0328) and larger family population (cOR = 1.13 (95% CI, 1.01–1.26), *P *= 0.0323) were the risk factors for MDR/RR isolates cluster, while high education (cOR =   0.25 (95%CI, 0.07–0.88), *P *= 0.0302) and occupation as non-physical workers (cOR =   0.44 (95% CI, 0.23–0.85), *P *= 0.0144) were the protective factors.

**Table 2.** Univariable analysis of risk factors associated with multidrug-resistance in genomic clusters.

Characteristics	Cluster rate	Crude OR	*P*-value
**Age**	** **		
<20	7 (35)	ref	
20–39	32 (24.24)	0.59 (0.22–1.62)	0.3084
40–59	44 (26.99)	0.69 (0.26–1.83)	0.453
60–79	32 (38.1)	1.14 (0.41–3.17)	0.7972
80+	2 (33.33)	0.93 (0.14–6.40)	0.94
**Gender**	** **		
Male	92 (30.77)	ref	
Female	25 (23.58)	0.69 (0.42–1.16)	0.1622
**Current residence**	** **		
Town	77 (29.96)	ref	
County	33 (26.83)	1.17 (0.72–1.89)	0.529
**Diabetes**	** **		
Yes	8 (23.53)	ref	
No	108 (29.27)	1.35 (0.59–3.06)	0.4809
**Hepatitis B**	** **		
Yes	1 (6.25)	ref	
No	104 (29.89)	6.39 (0.83–49.03)	0.0743
**Previous treatment**	** **		
Yes	32 (17.88)	ref	
No	85 (37.61)	2.77 (1.74–4.42)	<.0001
**Smear microscopy**			
Negative	12 (18.46)	ref	
Positive	105 (31.91)	2.07 (1.06–4.04)	0.0328
**Nationality**	** **		
Han	111 (29.21)	ref	
Other	6 (23.08)	0.73 (0.28–1.86)	0.5058
Education	** **		
Primary and below	49 (30.43)	ref	
Middle school	57 (30.32)	1.00 (0.63–1.57)	0.9813
University and above	3 (10)	0.25 (0.07–0.88)	0.0302
**Occupations**	** **		
Farmer	82 (33.33)	ref	
Student	6 (35.29)	1.09 (0.39–3.05)	0.8684
Non-physical workers	13 (18.06)	0.44 (0.23–0.85)	0.0144
Other (housekeeper or unemployed)	16 (22.54)	0.58 (0.31–1.08)	0.0852
**Family populations size**		1.13 (1.01–1.26)	0.0323

## Discussion

Halting TB transmission is a significant and challenging global priority worldwide [15]. Recently, *M. tuberculosis* WGS has been used to estimate recent transmission at a population level [16,17]. In this study, our approach involved the integration of WGS and epidemiological investigation on 5052 MTB isolates collected nationwide through cluster sampling. This strategy aimed to elucidate the transmission network of tuberculosis and MDR/RR tuberculosis in China.

In this cross-sectional study, the national clustering rate of isolates was 23.0%, a value lower than previous reports [9,18]. Possible reasons for this disparity include the low coverage rate of TB patients nationally, the lack of multi-year surveillance to date, and potential bias in sampling due to only sequencing culture-positive patients’ specimens (34.02% of total cases, 7,298/21,454). Increasing favourable culture rates, implementing direct-from-sputum sequencing, conducting continuous surveillance, and expanding surveillance scope are essential steps to construct a more comprehensive TB transmission network in China.

Overall, the cross-county clustering rate detected by this study is low, suggesting that tuberculosis transmission in China mainly occurs within the county. As such, the most significant public health benefit is expected from screening residents, identifying new tuberculosis patients, and strengthening patient management.

Our genomic epidemiological study of MTB isolates indicated that younger people have an increased risk of clustering, crowded living conditions and active social lives may contribute to this situation. A larger family size was also observed to be more likely to form clusters, indicating the household setting as an essential reservoir of *M. tuberculosis* transmission, and thus argues in favour of routine and extensive screening latency TB infection of the household contacts of TB patients.

Additionally, new tuberculosis cases were more prone to transmission of disease in comparison with relapsed cases, which might be because new cases were more likely to have a delayed diagnosis than relapsed cases [19], and delays in the diagnosis of tuberculosis or initiation of adequate treatment increase the prevalence of infectious tuberculosis, thereby also increasing the probability of recent transmission between individuals.

Furthermore, infection with an MDR/RR strain was also identified as a risk factor for tuberculosis transmission, which is consistent with previous studies [7,8], which may be partially due to untimely and inaccurate MDR/RR-TB diagnostics, as well as inadequate MDR/RR-TB patient management in China. Since most MDR/RR-TB cases remain undetected or are inappropriately managed, the transmission from their isolates will result in a much more severe MDR/RR-TB epidemic. As WHO recommended, access to universal drug susceptibility testing for all TB patients is urgently needed in China. We also found that patients with smear-positive results were more likely to contribute to recent transmission than those with smear-negative results [20,21]. These results highlight the importance of strict management in confirmed MDR patients with smear-positive until they are cured or show sputum smear conversion.

Interestingly, higher academic education qualifications were identified as a protective factor. This phenomenon maybe due to this population’s better health awareness and knowledge about protecting themselves.

Kendall et al. have demonstrated that current estimates of MDR-TB prevalence among TB notifications are most consistent with the hypothesis that over 80% of incident MDR-TB in present-day epidemic settings results from transmission of MDR-TB rather than selection of de novo resistance during previous treatment of the index case [22]. Chongguang Yang et al. also found that up to 72·5% (235/324, 95% CI 67.3%−77.3%) of MDR-TB patients were likely caused by transmission of MDR strains in Shanghai, China [7]. In this study, we observed that among at least 61.4% of the MDR isolates may be attributable to transmission in China. Considering the low coverage rate of TB patients nationally, the loss of transmission chain, and the surveillance time is short (TB usually takes up to 6–12 months after infection to cause noticeable disease), the transmission of MDR/RR-TB in China could be much more severe. Yang et al. have previously confirmed that patients with a delayed diagnosis or those older than 45 years were independently associated with genomic clusters of MDR-TB in Shanghai, China [7]. Our research found that previously treated, smear-positive, and larger family populations were independently associated with clustering MDR/RR-TB. High education and occupation as non-physical workers (including commercial service personnel, enterprise staff, medical personnel, educational staff, civil servant and public institutional person) were the protective factors for the MDR/RR-TB genomic cluster.

Several limitations should be acknowledged in this study. Firstly, the short study time frame (only one year) and low culture-positive rate may have led to the loss or disruption of some transmission chains. Secondly, the study’s retrospective nature and large study population resulted in the lack of a comprehensive epidemiological survey of the patients. This finding also includes the absence of contact investigations to confirm the linkages between the clustering isolates using epidemiological data and the locations they frequented where transmission could have occurred. Thirdly, the impact of delayed diagnosis on transmission was not assessed as relevant data was unavailable.

In summary, we used a combined genomic epidemiology approach to assess the clustering characteristics of TB and MDR/RR-TB at the population level in China and analyzed the risk factors associated with recent transmission. Furthermore, our findings highlight that the transmission of drug-resistant TB isolates plays a significant role in the burden of MDR/RR-TB in China. Urgent interventions, including early diagnosis of TB, rapid detection of drug resistance, and the implementation of strict isolation treatment strategies for high-risk populations and drug-resistant patients, are urgently needed to stem the epidemic of TB and drug-resistant TB in China.

## Supplementary Material

supplementary_tables

phylogenetic_tree_supplmentary_for_review

Supplementary_Figures
