# Supplementary figures and images for: Transmission characteristics in Tuberculosis by WGS: nationwide cross-sectional surveillance in China
Source: Emerg Microbes Infect. 2024 Apr 30;13(1):2348505. doi: 10.1080/22221751.2024.2348505 (PMC11097701; doi:10.1080/22221751.2024.2348505)

Tree scale: 0.1

Drug-resistant type

MDR-TB

XDR-TB

Lineage

Lineage 1

Lineage 2

Lineage 3

Lineage 4

M.bovis

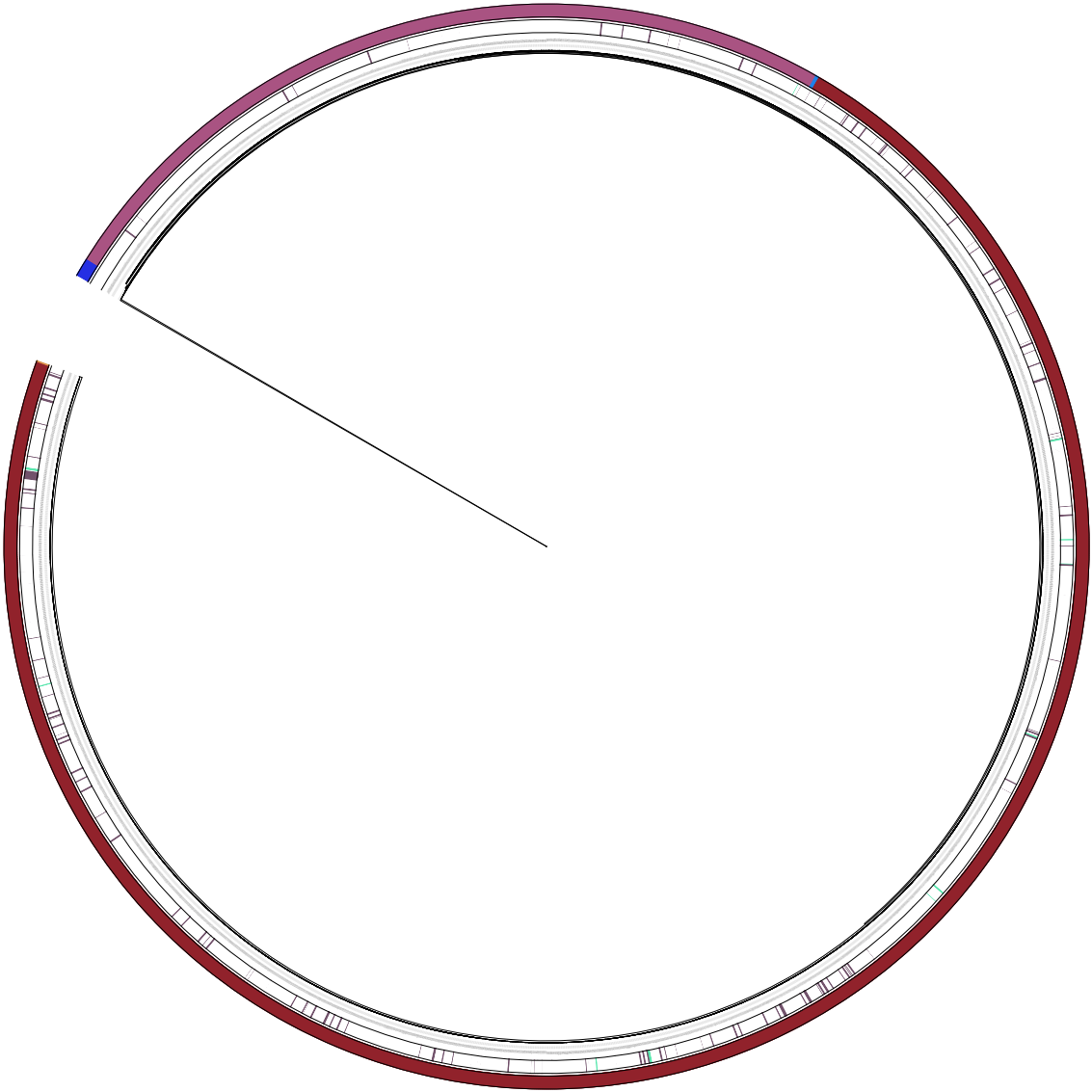

Supplement: phylogenetic_tree_supplmentary_for_review [file TEMI_A_2348505_SM5858.pdf]

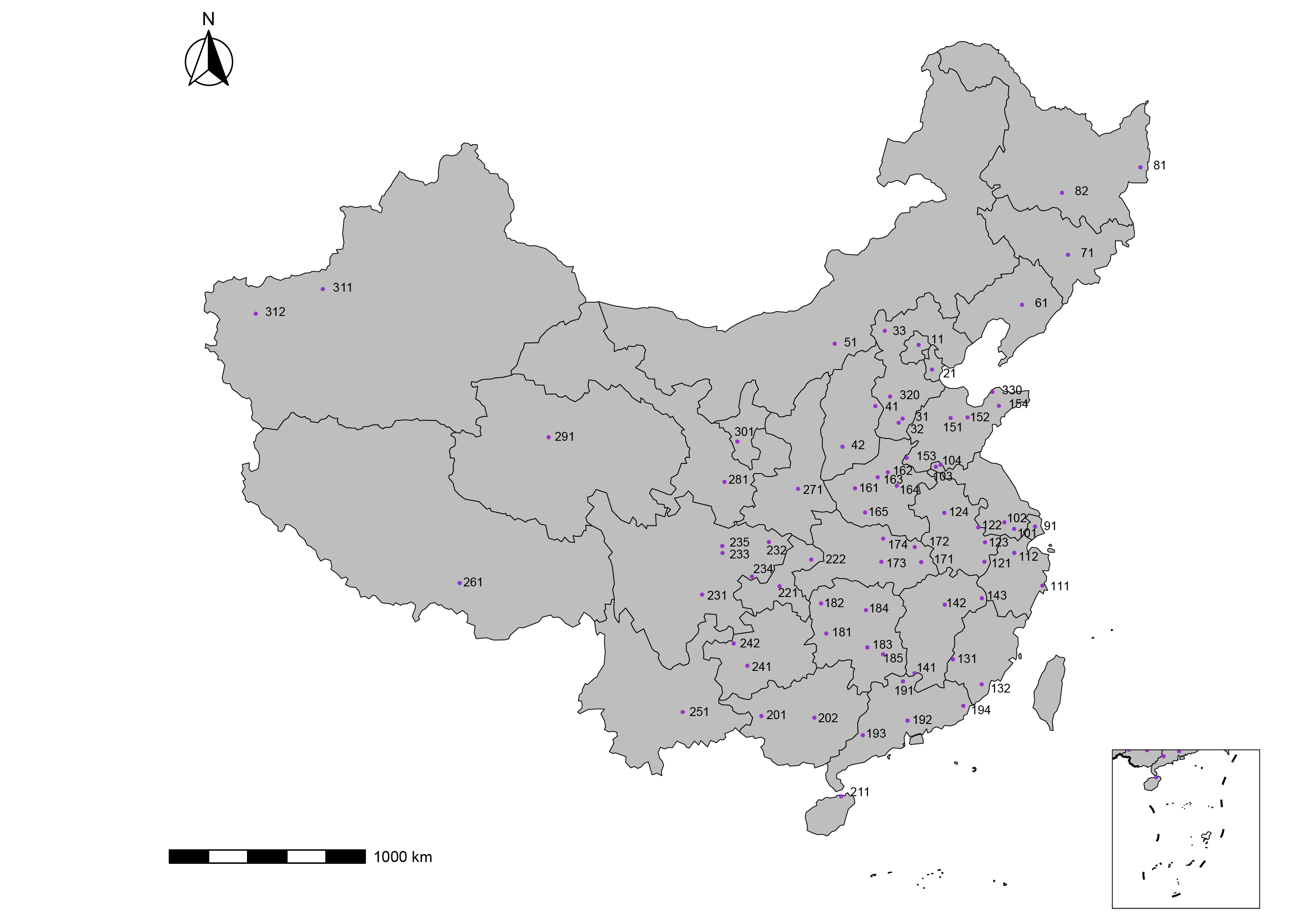

Supplement: Supplementary_Figures [file TEMI_A_2348505_SM5857.zip › Supplementary Figure 1.jpg]

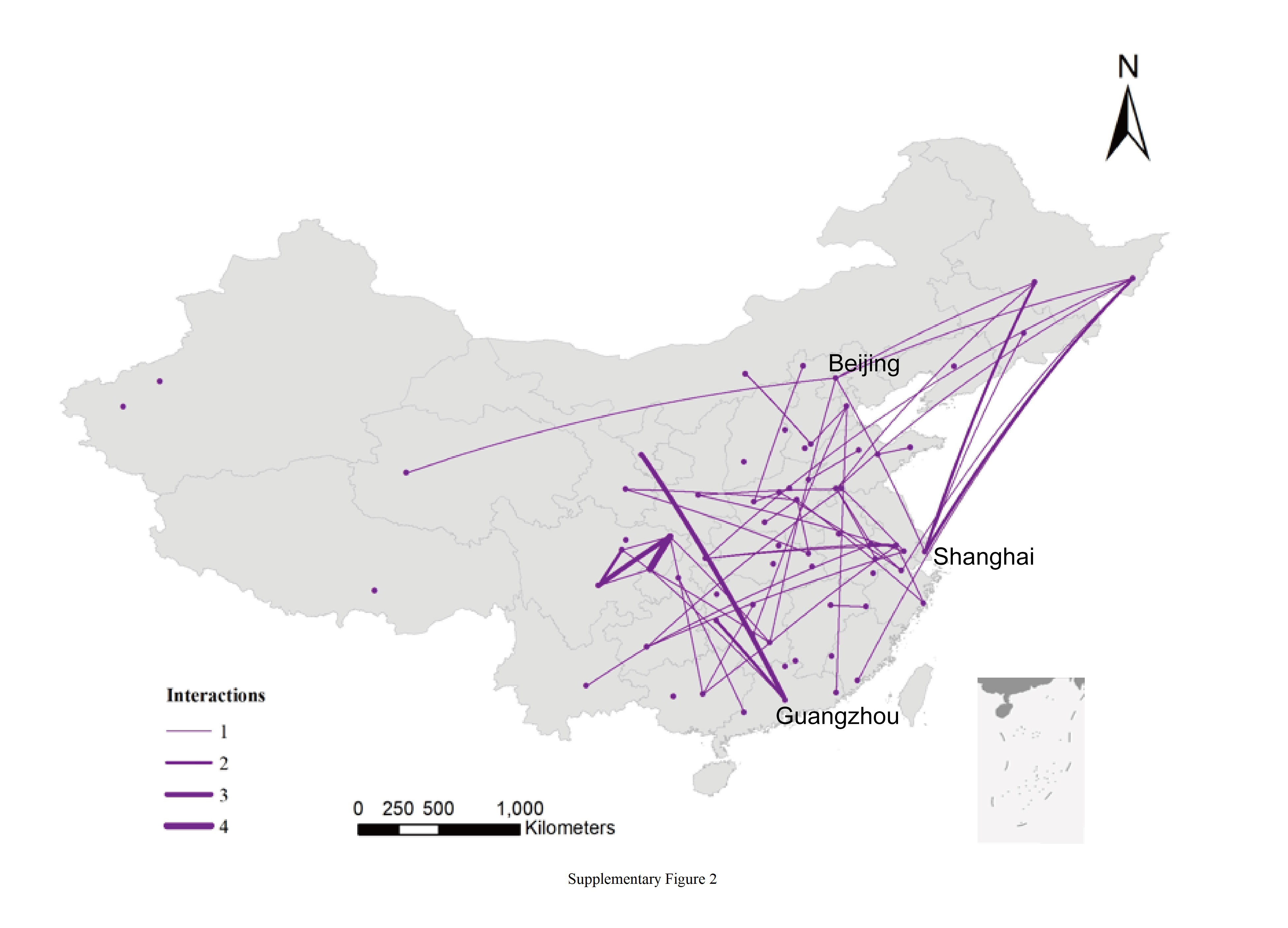

Supplement: Supplementary_Figures [file TEMI_A_2348505_SM5857.zip › Supplementary Figure 2.jpg]
